# Pesticide Poisoning and the Use of Personal Protective Equipment (PPE) in Indonesian Farmers

**DOI:** 10.1155/2020/5379619

**Published:** 2020-01-21

**Authors:** Tri Joko, Nikie A. Y Dewanti, Hanan L. Dangiran

**Affiliations:** Department of Environmental Health, Diponegoro University, Semarang 50271, Indonesia

## Abstract

This study aimed to investigate the poisoning symptoms occurring in pesticide-exposed farmers. This study was conducted in a red onion farming center area in Wanasari Subdistrict, Brebes, Indonesia, from May to June 2018. This study was designed as the descriptive study. The significance value of *p* < 0.005 showed that the variable was correlated with the health problems, including excessive fatigue (*p* value = 0.041), excessive saliva (*p* value = 0.006), hard breathing (*p* value = 0.021), frequent urination (*p* value = 0.047), blurred vision (*p* value = 0.059), dizziness (*p* value = 0.032), and finger pain (*p* value = 0.007). The significance value (*p* < 0.005) of 0.000 showed that the use of personal protective equipment was correlated with the health problems. Based on the odds ratio value of 1.137, 95% confidence interval = 0.042–0.444 meant that the personal protective equipment was a risk factor of health problems. The results showed that 89.2% of the farmers who used the personal protective equipment were categorized in the healthy group.

## 1. Introduction

Pesticides remain an integral part of agricultural activities worldwide. Pesticides are often used to increase productivity and quality of agricultural products. Pesticides are chemical compounds that are used to kill, repel, or control pests to protect plants before and after harvest. Pesticides work with damage to target organisms. But the way pesticides work is not specific to one species. The most widely used pesticides are organophosphate, carbamate, and pyrethroid insecticides [1]. The impact of pesticide exposure has become a globally developing environmental health problem. Increased vulnerability of farmers to pesticide poisoning is caused by a lack of knowledge about handling pesticides safely and appropriately [2, 3]. Even pesticide poisoning is one of the biggest problems in the world. The World Health Organization reports that there are at least around 18.2 per 100,000 agricultural workers who experience pesticide poisoning related to their work [4]. Pesticides can enter the body through inhalation, dermal absorption, and ingestion during preparation to applying pesticides. To reduce exposure to pesticides to protect health, farmers' use of PPE during the application of pesticides is recommended by the International Labor Organization (ILO) and the World Health Organization (WHO) [5]. Acute pesticide poisoning is generally underdiagnosed among farmers. This often happens in developing countries [6, 7].

Indonesia as an agricultural country has a high level of pesticide consumption. The data from the Indonesian government showed that, in 2013, Indonesia had an agricultural area of 8,112,103 hectares. Central Java was one of the provinces with the largest agricultural area in Indonesia. The area of agriculture in Central Java Province is 952,525 hectares. In addition, Brebes Regency is one of the centers of agriculture, especially onion horticultural. The area of the agricultural land in Brebes Regency is 60,341 hectares. Central Java Province gave the biggest contribution to onions in Indonesia by 42.70%. The regency with the largest production of shallots is Brebes, which produces 375,974 tons or 72.39% contributing to the total onion production in Central Java Province [8]. Onion plants are a type of plant that is very susceptible to pest attacks. The pesticides to control pests are not of only one type. Usually, farmers mix up more than three types of pesticides in one spray. Previous studies showed that the agricultural area in Wanasari Subdistrict, Brebes Regency, Indonesia, had chlorpyrifos residues >LOD. Even organophosphate pesticides with active ingredients as methidathion and malathion are also found in soil residues in agricultural areas [9]. This showed that pesticides are used in massive and continuous quantities, so the residue is left on the ground for even more than one to three months.

Pesticides that are widely used in Indonesia such as organophosphates, pyrethroids, and carbamates certainly have negative impacts on health. Organophosphate and carbamate pesticide compounds bind to cholinesterase. Phosphate radicals bind covalently to the active site of cholinesterase which creates a cholinergic effect on the central nervous system [10]. Organophosphate poisoning is even associated with increased lipid peroxidation and low levels of glutathione which causes damage to cell membranes and DNA [11]. Various cases of pesticide poisoning have been reported since many years. One of the biggest cases of pesticide poisoning is in Morocco. From 2008 to 2014, there were more than 2000 cases of acute poisoning [12]. A study showed that 50% of the pesticides used were from WHO classes I and II. From the results of the study, it was found there were 88% of respondents experiencing acute poisoning which was significantly related to the length of work. [13] A study conducted in Yogyakarta, Indonesia, showed health problems such as tremors in groups of farmers poisoned with organophosphate pesticides [14]. Organophosphate pesticide poisoning can be acute and chronic. Pesticide poisoning can cause various health problems. Some of them are muscarinic symptoms consisting of headache, blurred vision, chest pain, excessive sweating, and others [13].

Interestingly, farmers in Indonesia on average do not pay attention to the importance of using the personal protective equipment (PPE). But the PPE that is not used properly is very risky in acute poisoning. In Indonesia, there are still minimal data related to poisoning due to pesticide exposure. Therefore, this research needs to be done to determine the level of pesticide poisoning that occurs in farmers in Indonesia. The purpose of this study was to investigate the relationship between the use of PPE and pesticide poisoning in the onion farmers in Wanasari, Brebes.

## 2. Materials and Methods

This research was an observational research with cross-sectional design. This study was conducted from May to June 2018. The respondents of this study were 100 farmers. Data taken were symptoms of acute pesticide poisoning including excessive fatigue, eye irritation, itchy skin, excessive saliva, nausea, vomiting, burning in the esophagus, abdominal cramps, difficulty breathing, cough, getting tired quickly, frequent urination, getting thirsty often, increased hunger, weight loss, slow recovery, blurred vision, dizziness, getting tensed at the nape, tingling, rheumatic knee, and finger pain. Symptoms of acute pesticide poisoning were reported personally by respondents through a questionnaire given. The data on the use of pesticide patterns were also taken in this study through the focus group discussion (FGD) involving the leader of the farmer group. The collected data were processed in stages: editing, coding, data entry, and data cleaning. Data analysis was done using chi-square continuity correction. The odds ratio (OR) and 95% confidence interval (CI) were calculated to determine whether the use of PPE is a risk factor or not for each symptom.

## 3. Results and Discussion

This study was conducted in a red onion farming center area in Wanasari Subdistrict, Brebes District. Every year, Wanasari Subdistrict produces high-quality red onions and distributes to other areas in Indonesia. Geographically, Wanasari Subdistrict is located in Brebes District and has a flat topography. This subdistrict is passed by Pemali River which becomes the irrigation source of the agricultural lands.

The results of observation about and interview with the farmers showed the classification pattern of the excessive pesticide usage. In spraying, the farmers mixed at least 2-3 types of pesticides, which could even turn to 5–7 types. The dosage used was 30–40 ml for each type. The use of pesticides was adjusted to the attack of pests and diseases on red onion plants. The excessive use of pesticide became out of control on the explosion of pests, and when the pests attacked less, the use of pesticides also became less. In spraying, the farmers used a spray tank with a capacity of 15 or 17 liters. For 1000 m^2^, at least two tanks are needed for one spraying. If the farmers used pesticides with at least 3 mixtures of 30 ml of each type, then one tank consisting of 90 ml pesticide was sprayed. For 1000 m^2^, at least 180 ml of pesticide was sprayed. If this amount is counted in a year, it will show the amount of pesticides absorbed by the soil. In general, the farmers in Wanasari Subdistrict could use the mixture of around 3–5 types of pesticides with the dosage of 30–40 cc for each type of pesticides. The mixtures might vary between insecticides and fungicides, and even between the mixtures of the same insecticides. In spraying, the farmers used a spray tank with a capacity of 15 to 17 liters. The measurement used to spray the plants in 1000 m^2^ is 2 tanks. The spraying is usually done 3 to 4 times a day. The determination of pesticide dosage is based on the recommendation when there is a normal pest attack. However, if this pest attack increases, then the farmers will add the dosage of the pesticides based on the needs. It means that when there is an increase in the pest attack, the farmers will spray the pesticides with the dosage that is not as recommended and is likely to be excessive.

The results of FGD showed that the red onion farmers in Wanasari Subdistrict did not spray as recommended. According to the statements from the farmers, the volume of pesticides they used was not measured because the pests were resistant to the low pesticide volume. However, the use of pesticides was also adjusted to the attack of the pests. If the intensity of pest attack was high, the farmers used the pesticides in a great amount. Usually, when there was pest attack, the farmers mixed 3 to 5 types of pesticides, and the measurement used was the pesticide bottle cap, in which case the volume of each cap is 5 ml for the small bottle and 10 ml for the medium bottle. The pesticide dosage used by the farmers was 30 ml or three medium bottle caps. The red onion farmers in Wanasari Subdistrict often mix the pesticides with the volume of 30–40 ml, and some of them mix <30 ml of pesticide for 1 tank in each spraying. The tank volume was 14 liters on average. The mixture of pesticides that is not in accordance with the standard brings negative impacts on the environment. One of them is the pests that are resistant. When the red onion pests become resistant, the farmers will use a greater amount of pesticides. The continuous exposure of pesticides in a great amount could affect the health of the red onion farmers.

The use of high-dose pesticides done occasionally and frequently in every season will cause some damages, including the accumulated pesticide waste in the agricultural and water products, pollution in the agricultural environment (water, soil, and air), the decrease of productivity, and animal poisoning and human poisoning that will harm their health. The adverse effects of exposure to pesticides in this group can cause various health problems. It is related to their involvement in the agricultural activities, such as preparing for the spraying, including the mixing of pesticides, the washing of the tools/clothes used to spray, removing the grass from the plants, and looking for the pests, spraying the plants, and harvesting. The behavior of the farmers in using pesticides sometimes violates the rules. Besides the excessive dosage, the farmers often mixed some types of pesticides, arguing that it was to increase the poison power towards the pests. Those actions are actually very disadvantageous, as they will affect the health of the farmers and the surrounding environment.

### 3.1. The Use of PPE (Personal Protective Equipment) of Red Onion Farmers

The results of the study on 100 respondents involved in the farming activities showed that all respondents (100%) were not complete in using the personal protective equipment, as they did not wear boots, gloves, glasses, trousers, and long-sleeved clothes fully in every farming activity. Usually, the farmers only wore one or two kinds of PPE. For instance, they only wore hats (100%) and long-sleeved clothes (97%). The farmers did not use full PPE because the PPE is considered to disturb the farmers' activities in working and the farmers were not free to move and had difficulty in breathing.

### 3.2. The Health Problems of Red Onion Farmers

**Figure 1:**
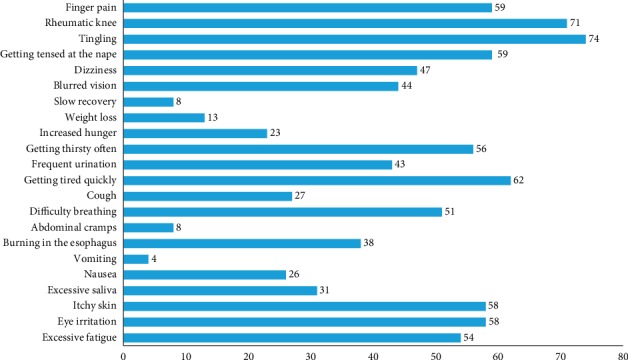
Pesticide poisoning symptoms reported by respondents.

The results of the study on 100 respondents involved in the farming activities showed that all respondents had health problems because they are often exposed to pesticides in their activities. The symptoms they often felt were tingling (74%), rheumatic knee (71%), getting tired quickly (62%), finger pain (59%), getting tensed at the nape (59%), eye irritation (58%), itchy skin (58%), getting thirsty often (56%), excessive fatigue (54%), difficulty breathing (51%), dizziness (47%), blurred vision (44%), frequent bowel movements (43%), burning in the esophagus (38%), excessive saliva (31%), cough (27%), nausea (26%), increased hunger (23%), weight loss (13%), abdominal cramps (8%), slow recovery (8%), and vomiting (4%) (Figure 1).

### 3.3. The Correlation between the Use of PPE and the Pesticide Poisoning Symptoms

There was a correlation between the use of PPE and the occurrence of health problems of red onion farmers in Wanasari Village, and a significance value (*p* < 0.005) was obtained which showed that the variable had a correlation with the occurrence of health problems. They were excessive fatigue (*p* value = 0.041), excessive saliva (*p* value = 0.006), hard breathing (*p* value = 0.021), frequent urination (*p* value = 0.047), blurred vision (*p* value = 0.059), dizziness (*p* value = 0.032), and finger pain (*p* value = 0.007). Based on the OR value > 1, 95% CI > 1 meant that the PPE is not a risk factor of health problems (Table 1).

Our results showed a significance value (*p* < 0.005) of 0.000 was obtained and that the use of PPE had a significant correlation with the occurrence of the health problems. Based on the OR value of 1.137, the 95% CI of 0.042–0.444 meant that the use of PPE is a risk factor of health problems. Based on the analysis, it was found that 74 farmers who used the PPE (89.2%) were categorized as healthy/not sick (Table 2).

Previous studies showed that symptoms of acute poisoning with the highest prevalence were headache, muscle pain, cough, weakness, eye pain, chest pain, and eye redness [15]. The prevalence of tiredness, fatigue, soreness in joints, thirst, and skin irritation shown in previous studies is a symptom of poisoning that is most often experienced by farmers [16].

The effect of pesticide exposure on the health depends on the type or active ingredients of the pesticides. The effect caused by the pesticides in the group of organophosphate-type malathion and chlorpyrifos in general is that an acute exposure to a high dose of pesticides can cause poisoning. The clinical indications of acute poisoning from the pesticides in the groups of organophosphate and carbamate are related to the excessive cholinergic stimulations such as fatigue, vomiting, nausea, diarrhea, headache, blurred vision, salivation, excessive sweat, anxiety, and respiratory failure. Meanwhile, the chronic poisoning is indicated by the cholinergic indications and the decrease of cholinesterase enzyme activities in the plasma, red blood cells, and brain. Organophosphate enters the body through skin absorption, inhalation, or ingestion. Furthermore, organophosphate compounds bind to acetylcholinesterase in red blood cells, making enzymes inactive. This condition causes excess acetylcholine in synapses and neuromuscular junctions. Excessive nicotinic stimulation can cause fasciculation and myoclonic jerks. Nicotinic receptors are also found in the adrenal glands which cause dry conditions. Organophosphate poisoning also produces symptoms associated with muscarinic receptor disorders [17–20].

The nicotinic acetylcholine (ACh) receptor (nAChR) is the main target of the insecticide. Nearly half of the insecticides used are neonicotinoids which act as nAChR or organophosphorus agonists and methylcarbamate acetylcholinesterase (AChE) inhibitors. Previous studies showed that analog or topical application of organophosphorus or methylcarbamate gives the same results as the inhibition and toxicity of nAChR in vivo, correlated with the inhibition of brain AChE in vivo. The conditions indicates that ACh is the organophosphorus or methylcarbamate induced nAChR active agent [21].

Pesticides sprayed into plants can enter the body through the respiratory tract and skin absorption. Agricultural work has been linked to rheumatic autoimmune diseases in different populations, and using different research designs, several studies showed the contribution of exposure to pesticides from agriculture to rheumatic autoimmune diseases [22]. Rheumatism is a multifactorial disease that is influenced by genetic and environmental risk factors [23]. Environmental risk factors associated with rheumatism are smoking and exposure to silica and pesticides [24–31]. Previous studies have not examined pesticides in rheumatic animal models, but the diverse immunotoxic effects seen in various types of pesticides support various mechanisms that can explain the hypothesis that pesticides contribute to rheumatism [32, 33]. Fonofos pesticides can change the level of methylation of genes involved in regulating the immune response [34]. Immunotoxic effects of other organophosphates have been described previously [32]. Carbamate and organophosphate pesticides have major toxicity mechanisms by inhibiting the activity of the acetylcholinesterase enzyme in neuronal and neuromuscular synapses [35]. The ability to inhibit the enzymes serine hydrolase and protease, which play an important role in the immune system, can have a general explanation for some changes in immune function caused by pesticides from organophosphate and carbamate groups [36–39].

The use of pesticides without using the correct PPE can apparently cause acute poisoning to farmers. Uncomfortable conditions in the body become an early sign that the body is not in good condition. So exposure to pesticides needs to be monitored to prevent the effects of poisoning that can interfere with health.

## 4. Conclusions

The use of PPE had a significant correlation with the pesticide poisoning symptoms and became a risk factor. The condition of pesticide poisoning found in this study could not be compromised. If this condition occurs continuously, it could have more serious health impacts. Due to the weaknesses of this study, subsequent studies can measure pesticides in blood or urine metabolites to confirm the occurrence of acute poisoning from pesticide exposure.

## Figures and Tables

**Table 1:** Correlation between the use of PPE and the occurrence of health problems of the red onion farmers in Wanasari Village.

Variable	Prevalence of sickness	*p* value	OR (95% CI)
Excessive fatigue			
Using PPE	41 (49.4%)	0.041	0.300 (0.042–0.444)
Using no PPE	13 (76.5%)		
Eye irritation			
Using PPE	45 (54.2%)	0.090	0.364 (0.110–1.211)
Using no PPE	13 (76.5%)		
Itchy skin			
Using PPE	47 (56.6%)	0.539	0.712 (0.241–2.108)
Using no PPE	11 (64.7%)		
Excessive saliva			
Using PPE	21 (25.3%)	0.006	0.237 (0.080–0.702)
Using no PPE	10 (58.8%)		
Nausea			
Using PPE	21 (25.3%)	0.725	0.813 (0.256–2.579)
Using no PPE	5 (29.4%)		
Vomiting			
Using PPE	2 (2.4%)	0.073	0.185 (0.024–1.418)
Using no PPE	2 (11.8%)		
Burning in the esophagus			
Using PPE	29 (34.9%)	0.164	0.477 (0.166–1.369)
Using no PPE	9 (52.9%)		
Abdominal cramps			
Using PPE	5 (6.0%)	0.108	0.299 (0.064–1.396)
Using no PPE	3 (17.6%)		
Difficulty breathing			
Using PPE	38 (45.8%)	0.021	0.260 (0.078–0.863)
Using no PPE	13 (76.5%)		
Cough			
Using PPE	21 (25.3%)	0.398	0.621 (0.204–1.886)
Using no PPE	6 (35.3%)		
Getting tired quickly			
Using PPE	51 (61.4%)	0.801	0.869 (0.293–2.582)
Using no PPE	11 (64.7%)		
Frequent urination			
Using PPE	32 (38.6%)	0.047	0.342 (0.115–1.016)
Using no PPE	11 (64.7%)		
Getting thirsty often			
Using PPE	45 (54.2%)	0.427	0.646 (0.218–1.910)
Using no PPE	11 (64.7%)		
Increased hunger			
Using PPE	19 (22.9%)	0.955	0.965 (0.281–3.308)
Using no PPE	4 (23.5%)		
Weight loss			
Using PPE	10 (12.0%)	0.532	0.639 (0.156–2.622)
Using no PPE	3 (17.6%)		
Slow recovery			
Using PPE	6 (7.2%)	0.530	0.584 (0.107–3.177)
Using no PPE	2 (11.8%)		
Blurred vision			
Using PPE	33 (39.8%)	0.059	0.360 (0.121–1.068)
Using no PPE	11 (64.7%)		
Dizziness			
Using PPE	35 (42.2%)	0.032	0.304 (0.098–0.941)
Using no PPE	12 (70.6%)		
Getting tensed at the nape			
Using PPE	46 (55.4%)	0.108	0.383 (0.115–1.272)
Using no PPE	13 (76.5%)		
Tingling			
Using PPE	59 (71.1%)	0.142	0.328 (0.070–1.544)
Using no PPE	15 (88.2%)		
Rheumatic knee			
Using PPE	56 (67.5%)	0.086	0.277 (0.059–1.297)
Using no PPE	15 (88.2%)		
Finger pain			
Using PPE	44 (53.0%)	0.007	0.150 (0.032–0.700)
Using no PPE	15 (88.2%)		

**Table 2:** Correlation between the use of PPE and the occurrence of health problems of the red onion farmers in Wanasari Village.

Variable of PPE usage	Health problem		Total
	Sick	Not sick	
Using PPE	9 (10.8%)	74 (89.2%)	83 (100%)
Using no PPE	8 (47.1%)	9 (52.9%)	17 (100%)
Total	17 (17.0%)	83 (83.0%)	100 (100%)
*p* value 0.0001; 95% CI 1.137 (min 0.042; max 0.444)			

## Data Availability

The data related to pesticide poisoning reports by respondents used to support the findings of this study are available from the corresponding author upon request.
